# Prevalence and impact of early prone position on 30-day mortality in mechanically ventilated patients with COVID-19: a nationwide cohort study

**DOI:** 10.1186/s13054-022-04122-w

**Published:** 2022-09-04

**Authors:** Lars Engerström, Johan Thermaenius, Johan Mårtensson, Anders Oldner, Johan Petersson, Jessica Kåhlin, Emma Larsson

**Affiliations:** 1 Department of Anesthesiology and Intensive Care and Department of Medical and Health Sciences, Linköping University, Norrköping, Sweden; 2 Department of Thoracic and Vascular Surgery and Department of Medical and Health Sciences, Linköping University, Linköping, Sweden; 3 The Swedish Intensive Care Registry, Region Värmland, Sweden; 4 Department of Physiology and Pharmacology, Karolinska Institutet, Stockholm, Sweden; 5 Department of Perioperative Medicine and Intensive Care, Karolinska University Hospital, 171 76 Stockholm, Sweden

**Keywords:** Prone position, Critical illness, COVID-19, Ventilation, Outcome, Mortality, ARDS

## Abstract

**Background:**

COVID-19 ARDS shares features with non-COVID ARDS but also demonstrates distinct physiological differences. Despite a lack of strong evidence, prone positioning has been advocated as a key therapy for COVID-19 ARDS. The effects of prone position in critically ill patients with COVID-19 are not fully understood, nor is the optimal time of initiation defined. In this nationwide cohort study, we aimed to investigate the association between early initiation of prone position and mortality in mechanically ventilated COVID-19 patients with low oxygenation on ICU admission.

**Methods:**

Using the Swedish Intensive Care Registry (SIR), all Swedish ICU patients ≥ 18 years of age with COVID-19 admitted between March 2020, and April 2021 were identified. A study-population of patients with PaO_2_/FiO_2_ ratio ≤ 20 kPa on ICU admission and receiving invasive mechanical ventilation within 24 h from ICU admission was generated. In this study-population, the association between early use of prone position (within 24 h from intubation) and 30-day mortality was estimated using univariate and multivariable logistic regression models.

**Results:**

The total study cohort included 6350 ICU patients with COVID-19, of whom 46.4% were treated with prone position ventilation. Overall, 30-day mortality was 24.3%. In the study-population of 1714 patients with lower admission oxygenation (PaO_2_/FiO_2_ ratio ≤ 20 kPa), the utilization of early prone increased from 8.5% in March 2020 to 48.1% in April 2021. The crude 30-day mortality was 27.2% compared to 30.2% in patients not receiving early prone positioning. We found no significant association between early use of prone positioning and survival.

**Conclusions:**

During the first three waves of the COVID-19 pandemic, almost half of the patients in Sweden were treated with prone position ventilation. We found no association between early use of prone positioning and survival in patients on mechanical ventilation with severe hypoxemia on ICU admission. To fully elucidate the effect and timing of prone position ventilation in critically ill patients with COVID-19 further studies are desirable.

**Supplementary Information:**

The online version contains supplementary material available at 10.1186/s13054-022-04122-w.

## Introduction

The current Coronavirus disease (COVID-19) pandemic has affected healthcare worldwide with large numbers of critically ill patients, where the surge of patients often outnumbered intensive care unit (ICU) resources. SARS-CoV-2 induces a large variation of symptoms, from mild catarrhalia to severe acute respiratory distress syndrome (ARDS) with requirement for invasive mechanical ventilation. Early prone position ventilation is an established intervention in patients with ARDS [1] leading to improved ventilation-perfusion matching and survival. Even so, prone position continues to be underused in moderate to severe ARDS [2, 3]. Recent studies during the COVID-19 pandemic indicate a striking increase in the use of prone position, despite scarce ICU resources [4, 5].

Interestingly, observational studies have found an association between the oxygenation response to prone positioning and improved outcome for COVID-19 ARDS [6, 7]. This contradicts previous findings in patients with non-COVID-19 ARDS where such an association is missing [8]. ARDS caused by COVID-19, as opposed to other etiologies, is reported to be more severe, display a lower PaO_2_/FiO_2_ ratio but with a greater lung compliance [6]. However, other studies reported a similar compliance in COVID-19 and non-COVID-19 ARDS [9]. Despite a lack of strong outcome evidence in COVID-19 ARDS prone positioning has been advocated as a key therapy in COVID-19 ARDS [5, 10–12].

The beneficial physiological effects of prone position with improved ventilation-perfusion matching and more homogenous inflation which may lead to less stress and strain and reduced inspiratory pressures are well established, but there is lack of evidence of whether this results in an overall survival improvement in invasively mechanically ventilated patients with COVID-19.

Few population-based reports have described the utilization of prone position in critically ill patients with COVID-19. Previous studies primarily describe the first wave of COVID-19 and with conflicting results on mortality benefit from early prone position [13–15]. Thus, uncertainty prevails regarding the association between prone position, including time of initiation, and outcome. In this nationwide cohort study, we aimed to investigate the use of prone position ventilation and to evaluate the overall effect on mortality of early initiation (within 24 h from intubation) of prone position in a study-population of mechanically ventilated COVID-19 patients with a PaO_2_/FiO_2_ ratio ≤ 20 kPa on admission.

## Material and methods

The Swedish Ethical Review Authority approved the study (approval number 2020-01477) and waived requirement for informed consent. The study adhered to the STROBE (Strengthening the Reporting of Observational Studies in Epidemiology) guidelines for cohort studies [16]. All research was conducted in accordance with national guidelines and regulations.

### Study design and population

All public health care in Sweden, including intensive care, is tax-funded and available for all citizens regardless of private health insurances. In co-operation with the Public Health Agency of Sweden, mandatory surveillance data of COVID-19 are routinely reported to the Swedish Intensive Care Registry (SIR). SIR is collecting individual patient data within the legal framework of the Swedish National Quality Registries. Written informed consent is not required, but patients have the possibility to withdraw their data from SIR at any time. Available data include baseline demographics, comorbidities, variables included in the Simplified Acute Physiology Score (SAPS3) and variables on process of care in the ICU. Data are transferred to SIR after local validation. After central validation at SIR, incomplete or inconsistent (entries outside pre-specified limits) data are returned to the specific ICUs for correction before data are added to the SIR database. All Swedish citizens have a unique personal identity number making linkage possible to the Swedish Population Register, and thereby ascertain mortality data. The personal identity number also enables analyses of readmissions and to follow the care of a patient between different ICUs.

In this nationwide cohort study, we identified all Swedish ICU patients ≥ 18 years of age with confirmed SARS-CoV-2 by polymerase chain reaction admitted between March 6, 2020, and April 30, 2021. Exclusion criteria included SARS-CoV-2-RNA positive patients with other reason for admission than COVID-19 and missing data on follow up (due to temporary personal identity number).

The main aim was to investigate the association between early use of prone position (within 24 h from intubation) and 30-day mortality. For these analyses we identified a study population with the following inclusion criteria: patients with a PaO_2_/FiO_2_ ratio ≤ 20 kPa within one hour before until one hour after arrival to ICU (registered for SAPS3 calculation) and receiving invasive mechanical ventilation within 24 h from ICU admission.

### Covariates and outcome

Baseline characteristics, including comorbidities, were defined at the time of ICU admission. Physiological variables were recorded on admission within one hour before until one hour after arrival to ICU. Within this time interval, for each parameter the worst was included in the analyses. Information on process of care includes the entire ICU stay. Thirty- and 90-day mortality were defined as mortality (all-cause) within 30 and 90 days from admission to ICU, respectively. Primary outcome was 30-day mortality and secondary outcome was 90-day mortality.

### Statistical analysis

Categorical variables are presented as number with percentage. Continuous variables are presented as median with interquartile range (IQR). Time to death was displayed using the Kaplan–Meier methodology. Normal distribution was assessed for the variable age with the Shapiro-Wilks test and revealed that age was significantly different from normal distribution.

For patients with a PaO_2_/FiO_2_ ratio ≤ 20 kPa on ICU admission and receiving invasive mechanical ventilation within 24 h from ICU admission, the association between early use of prone position (within 24 h from intubation) and 30-day mortality was estimated using univariate and multivariable logistic regression models and expressed as odds ratios (OR) with corresponding 95% confidence intervals. A priori selected covariates including patient sex, age, comorbidities (cardiac disease, chronic obstructive pulmonary disease (COPD)/asthma, obesity (BMI > 40 kg/m^2^), hypertension, immune deficiency, chronic liver disease, chronic kidney disease, neuromuscular disease and malignancy (neoplasia spread beyond regional lymph nodes)), PaO_2_/FiO_2_ ratio, SAPS3 and admission period. To avoid collinearity, age, comorbidity and PaO_2_/FiO_2_ ratio components were removed from SAPS3. All variables in the univariate models were included in the multivariable models. Propensity score matching and inverse probability of treatment weighting (ipw) are methods that aim to achieve a balanced distribution of confounders between treatment groups more like a randomized trial, having advantages over traditional regression models [17]. We also performed ipw analysis with early prone as treatment and 30-day mortality as outcome and included the above-mentioned predictors as covariates to investigate. We evaluated the balance of the covariates between the weighted treatment groups by standardized mean difference and used a robust (sandwich) variance estimator. Furthermore, we performed a logistic regression model exploring 90-day mortality. To test the robustness of our findings we performed logistic regression models exploring 30-day mortality in the following two subgroups 1) patients with PaO_2_/FiO_2_ ratio ≤ 13.3 kPa on ICU admission and 2) patients still in treated in ICU within 48 h from ICU admission.

In addition, the study population was divided into two admission years, 2020 and 2021 and compared with respect to process of care. Utilization of early prone position over time was visualized with a Loess smoothed plot.

For patients with more than one ICU admission, ICU data from all admissions were included in the analyses. Data were analyzed as complete cases. A p-value of 0.05 was considered statistically significant. All data were analysed using R 4.1.1 (R Core Team (2021). R Foundation for Statistical Computing, Vienna, Austria) and Stata/SE 16 (StataCorp, Collage Station, TX, USA).

## Results

### Patients

**Fig. 1:**
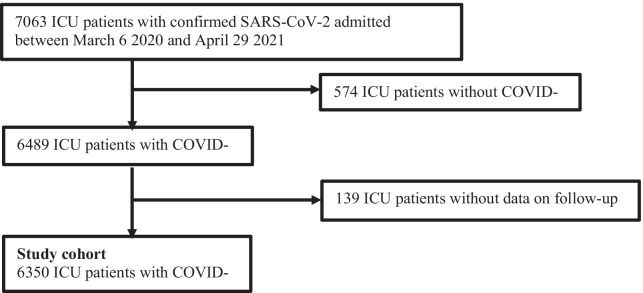
Flow chart of included patients

From March 6, 2020, to April 30, 2021, a total of 7063 ICU patients with confirmed SARS-CoV-2 were reported to SIR. We excluded 574 patients with a primary diagnosis not associated with COVID-19 and 139 patients without data on follow-up (patients who are not Swedish residents and receive temporary patient numbers and patients emigrating during the study), yielding a total study cohort of 6350 ICU patients with COVID-19 (Fig. 1).

Approximately half of the patients (47.4%) were treated with prone position ventilation (Additional file 2: Table S1a).

Patients in the total study cohort never receiving prone positions ventilation during ICU stay are hereafter referred to as “prone = no” and patients receiving prone position as “prone = yes”.

**Fig. 2:**
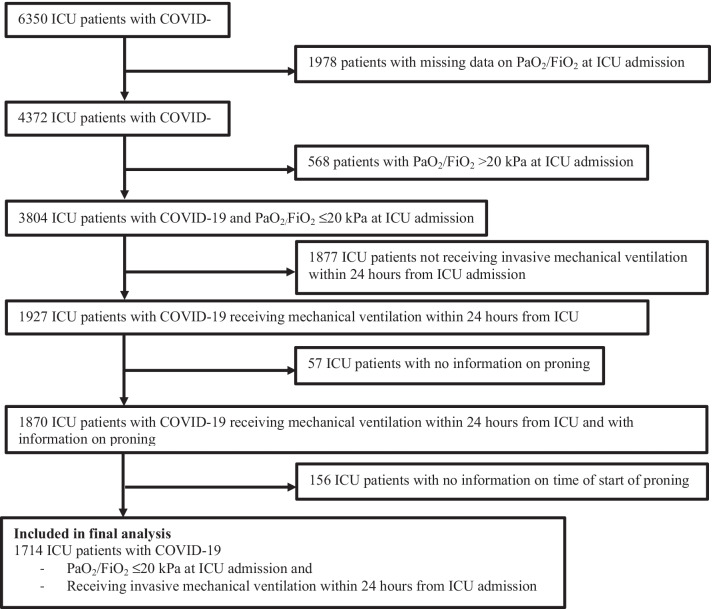
Identification of patients with early mechanical ventilation and PaO2/FiO2 ≤ 20 kPa at ICU admission

From the total study cohort, we extracted a study-population of patients with PaO_2_/FiO_2_ ratio ≤ 20 kPa on ICU admission *and* receiving invasive mechanical ventilation within 24 h from ICU admission. To generate this study-population the following patients were excluded from the total study cohort: 1978 patients with no data on PaO_2_/FiO_2_ ratio on ICU admission, 568 patients with PaO_2_/FiO_2_ > 20 kPa on ICU admission, 1877 patients who did not received invasive mechanical ventilation within 24 h from ICU admission, 57 patients with no information on proning and 156 patients with no information on time of start of proning (Fig. 2).

Thus, 1714 ICU patients were identified in this study-population and included in the logistic regression models of 30- and 90-day mortality. Patients within the study-population who were not treated with prone position initiated within 24 h from start of invasive mechanical ventilation are hereafter referred to as “early prone = no” and patients treated with prone position within 24 h from start of invasive mechanical ventilation as “early prone = yes”.

### Total study cohort—baseline characteristics

Of the total cohort 6350 patients, 1843 (29.0%) were women. Median age was 64 (IQR 55–72) years. For 393 (6.2%) of the patients there were no data on proning. Most of the patients were admitted from hospital floor (77.6%). Median duration of symptoms before ICU admission was 10 (IQR 7–13) days. Approximately one quarter (27.8%) of the patients had no reported comorbidity on admission. Baseline characteristics are presented in detail in Additional file 2: Table S1a.

### Total study cohort—process of care

Median total length of ICU stay was 10 (IQR 4–19) days; 5 (IQR 2–11) days for “prone = no” and 15 (IQR 9–26) days for “prone = yes”. Overall, 4174 patients (65.7%) patients received invasive mechanical ventilation. The median total duration of invasive mechanical ventilation was 11.6 (IQR 6.2–20.4) days. The median time from ICU-admission to start of invasive mechanical ventilation was 4.0 h and the median time from ICU-admission to treatment in prone position was 20.4 h. Renal replacement therapy was reported in 771 (13.5%) of the patients, 55 (1.6%) patients received extra corporeal membrane oxygenation (ECMO) and 1562 (24.6%) patients underwent tracheostomy. Care provided in the ICU for the total study cohort is presented in Additional file 2: Table S2.

As shown in Fig. 2, 1978 (31.1%) patients had missing data on PaO_2_/FiO_2_ ratio on ICU admission. A majority of these patients did not receive mechanical ventilation on admission, and under these circumstances FiO_2_ is not routinely reported to SIR. The median age among these patients was 64 (IQR 54–72) years, 542 (27.4%) were women and 393 (19.8%) patients had died within 30 days from ICU admission.

*Patients with PaO*_*2*_*/FiO*_*2*_* ratio* ≤ *20 kPa on ICU admission and receiving invasive mechanical ventilation within 24 h from ICU admission—baseline characteristics for the study population.*

Baseline characteristics are presented in Tables 1 and 2. Of the 1714 patients, 512 (29.9%) were women and the median age was 64 (IQR 55–71) years. Approximately one-third (28.8%) had no reported comorbidity on admission; 356 (31.1%) and 137 (24.0%) for “early prone = no” and “early prone = yes”, respectively. Median PaO_2_/FiO_2_ ratio on ICU admission was 11.6 (IQR 8.8–14.8) kPa for “early prone = no” and 9.9 (IQR 8.0–12.6) kPa for “early prone = yes”. For “early prone = no”, median SAPS 3 score was 58 (IQR 53–65) and “early prone = yes” was 58 (IQR 53–66).

**Table 1:** Characteristics for patients with PaO_2_/FiO_2_ ≤ 20 kPa on invasive mechanical ventilation within 24 h from ICU admission

	All	Early prone = NO	Early prone = YES
*Patient demographics, characteristics and comorbidities at ICU admission*			
No. (%)	1714	1144 (66.7)	570 (33.3)
Women	512 (29.9)	346 (30.2)	166 (29.2)
Age, median (IQR), y	64 (55–71)	64 (55–71)	64 (54–71)
*Age, Interval, y*			
< 40	85 (5)	61 (5.3)	24 (4.2)
40–49	175 (10.2)	117 (10.2)	58 (10.2)
50–59	387 (22.6)	254 (22.2)	133 (23.3)
60–69	549 (32)	362 (31.6)	187 (32.8)
70–79	459 (26.8)	309 (27)	150 (26.3)
≥ 80	59 (3.4)	41 (3.6)	18 (3.2)
*Admission month*			
March – April 2020	514 (30)	426 (37.2)	88 (15.4)
May – August 2020	228 (13.3)	162 (14.2)	66 (11.6)
September – December 2020	326 (19)	203 (17.7)	123 (21.6)
January – April 2021	646 (37.7)	353 (30.9)	293 (51.4)
*Location before ICU admission*			
Emergency department	398 (23.2)	242 (21.2)	156 (27.4)
Hospital floor	1316 (76.8)	902 (78.8)	414 (72.6)
*Time from symptom to ICU admission, median (IQR), d*			
No. with data	1688	1124	564
Median (IQR), d	10 (7–13)	10 (7–13)	10 (8–14)
*Hospital level*			
Tertiary	576 (33.6)	376 (32.9)	200 (35.1)
County	964 (56.2)	636 (55.5)	328 (57.5)
Local	174 (10.2)	132 (11.5)	42 (7.4)
*Days at hospital before ICU admission*			
Median (IQR), d	2 (0–4)	2 (0–4)	2 (0–5)
Pregnant, No	16	14	2
*Comorbidities*			
None	493 (28.8)	356 (31.1)	137 (24.0)
One or more	1221 (71.2)	788 (68.9)	433 (76.0)
Chronic hypertension	825 (48.1)	521 (45.5)	304 (53.3)
Chronic cardiac disease	239(13.9)	162(14.2)	77 (13.5)
COPD/Asthma	275 (16.0)	178(15.6)	97(17)
Immune deficiency	130 (7.6)	77 (6.7)	53 (9.3)
Chronic liver disease	12 (0.7)	7 (0.6)	5 (0.9)
Chronic kidney disease	97 (5.7)	69 (6.0)	28 (4.9)
Diabetes	484 (28.2)	321 (28.1)	163 (28.6)
Neuromuscular disease	18 (1.1)	10 (0.9)	8 (1.4)
Obesity^a^	188 (11.0)	111 (9.7)	77 (13.5)
Malignancy^b^	31 (1.8)	21 (1.8)	10 (1.8)

IQR, interquartile range; y: years; d: days; ICU, intensive care unit; COPD, chronic obstructive pulmonary disease

^a^Obesity is defined as BMI > 40 kg/m^2^

^b^Malignancy is defined as neoplasia spread beyond regional lymph nodes

**Table 2:** ICU-parameters for patients with PaO_2_/FiO_2_ ≤ 20 kPa on invasive mechanical ventilation within 24 h from ICU admission

***Ventilatory parameters at ICU admission (within one hour before until one hour after admission)***			
PaO_2_, kPa. Median (IQR)	8.5 (7.4–9.8)	8.6 (7.4–9.9)	8.3 (7.3–9.4)
FiO_2_, %. Median (IQR)	80 (70–100)	80 (65–95)	85 (70–100)
PaO_2_/FiO_2_ ratio. Median (IQR)	11.0 (8.5–14.2)	11.6 (8.8–14.8)	9.9 (8.0–12.6)
*PaO*_*2*_*/FiO*_*2*_* ratio categories. kPa*			
> 13.3—≤ 26.6	525 (30.6)	415 (36.3)	110 (19.3)
≤ 13.3	1189 (69.4)	729 (63.7)	460 (80.7)
***Treatment and vital signs at ICU admission (within one hour before until one hour after admission)***			
Vasopressor on admission, No (%)	107 (6.2)	73 (6.4)	34 (6.0)
*Body temperature*			
No with data	1647	1094	553
Degrees Celsius	37.8 (37.0–38.4)	37.8 (37.0–38.5)	37.8 (37.1–38.3)
Fever^a^, No (%)	602 (36.6)	423 (38.6)	180 (32.5)
*Systolic blood pressure, min, mmHg*			
No. with data	1669	1103	566
Median (IQR)	119 (95–135)	116 (95–134)	120 (100–139)
*Heart rate, maximum, beats/min*			
No. with data	1681	1116	565
Median (IQR)	95 (82–110)	95 (80–110)	98 (84–112)
*White blood cell count,* × *10*^*9*^*/L*			
No. with data	1638	1084	554
Median (IQR)	9.3 (6.8–12.9)	9.0 (6.5–12.6)	9.9 (7.3–13.1)
*pH*			
No. with data	1699	1130	569
Median (IQR)	7.43 (7.35–7.47)	7.43 (7.35–7.47)	7.44 (7.36–7.48)
*Creatinine, mg/dL*			
No. with data	1636	1085	551
Median (IQR)	72 (58–95)	71 (58–99)	72 (58–91)
*Bilirubin, mg/dL*			
No. with data	1596	1047	549
Median (IQR)	9 (6–12)	9 (6–12)	9 (6–12)
SAPS3 at admission, median (IQR)	58 (53–65)	58 (53–65)	58 (53–66)
Predicted risk of death (SAPS3), median (IQR), %	32 (22–48)	32 (22–46)	32 (22–48)
***Outcome***			
30-day mortality	500 (29.2)	345 (30.2)	155 (27.2)
90-day mortality	575 (33.5)	398 (34.8)	177 (31.1)

IQR, interquartile range; y: years; d: days; ICU, intensive care unit; PaO_2,_ arterial partial pressure of oxygen; FiO_2_, fraction of inspired oxygen; SAPS, simplified acute physiology score

^a^Fever is defined as body temperature above 38 °C

### Patients with PaO_2_/FiO_2_ratio ≤ 20 kPa on ICU admission and receiving invasive mechanical ventilation within 24 h from ICU admission—process of care for the study population

The use of early prone increased from 8.5% in March 2020 to 48.1% in April 2021. The median total duration of invasive mechanical ventilation was 11.0 (IQR 5.7–18.8) days for patients with “early prone = no” and 11.2 (6.9–18.5) days for patients with “early prone = yes”. Renal replacement therapy was reported in 20.2% and 16.7% for “early prone = no” and “early prone = yes”, respectively. Median total length of ICU stay was 14 (IQR 8–22) days for “early prone = no” and 14 (IQR 9–22) days for “early prone = yes”. For “early prone = yes”, patients who died within 30 days had a median length of ICU stay of 13 (IQR 8–18) days, corresponding figure for patients who survived 30 days was 15 (IQR 9–19) days. For “early prone = no”, patients who died within 30 days had a median length of ICU stay of 11 (IQR 6–12) days, corresponding figure for patients who survived 30 days was 15 (IQR 9–19) days. Care provided in the ICU for the study-population is presented in Table 3.

**Table 3:** ICU care provided for patients in study-population with low oxygenation and early mechanical ventilation

Variable	No (%)		
	All (n = 1714)	Early prone = NO (n = 1144)	Early prone = YES (n = 570)
Duration of invasive mechanical ventilation, median (IQR) h	267 (143–450)	265 (137–452)	269 (165–444)
Renal replacement therapy, No./total (%)	312/1638 (19.0)	219/1082 (20.2)	93/556 (16.7)
ECMO, No./total (%)	23/982 (2.3)	17/660 (2.6)	6/322 (1.9)
Tracheostomy, No. (%)	578 (33.7)	389 (34.0)	189 (33.2)
More than one ICU admission^a^	576 (33.6)	408 (35.7)	168 (29.5)
ICU length of stay, median (IQR), d	14 (8–22)	14 (8–22)	14 (9–22)

IQR, interquartile range; ECMO, extracorporeal membrane oxygenation; h, hours; ICU, intensive care unit; d, days

^a^Most often due to optimization of ICU resources

Utilization of early prone position over time is displayed in Additional file 1: Fig. S1. Process of care for the study population divided into two periods of the COVID-19 pandemic (2020 and 2021) is displayed in Additional file 2: Table S6.

### Mortality

**Fig. 3:**
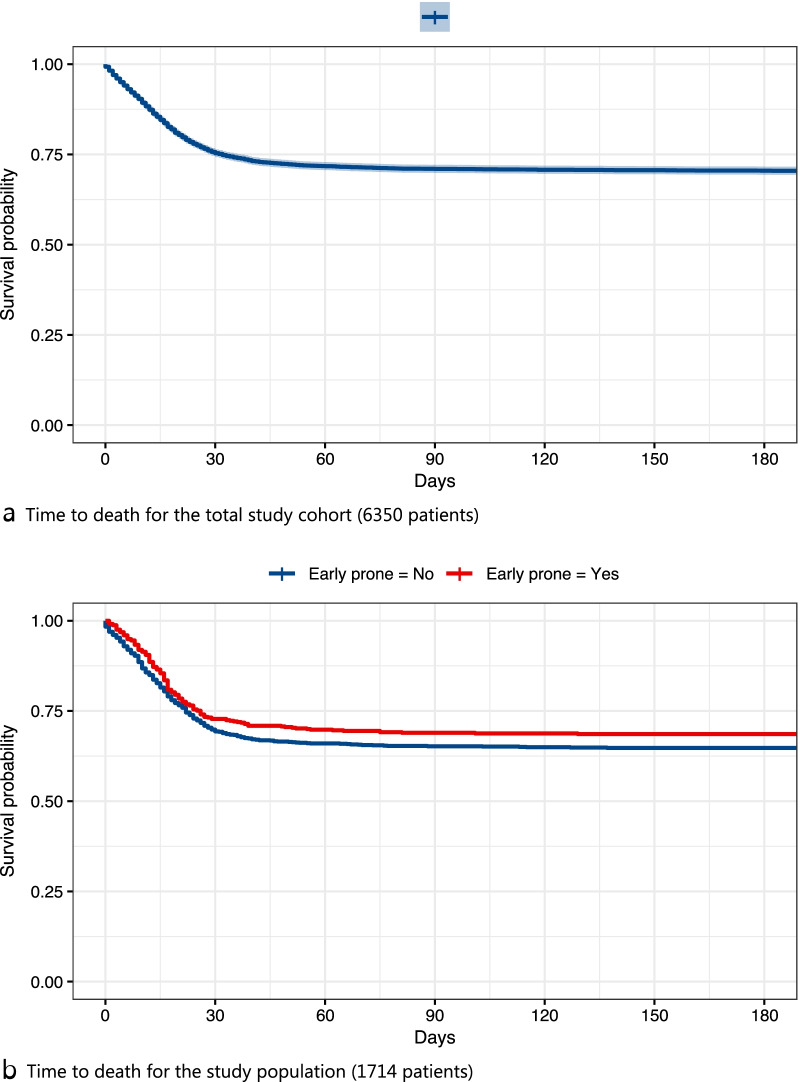
Time to death for the total study cohort and the study population **a** Time to death for the total study cohort (6350 patients) **b** Time to death for the study population (1714 patients)

Overall time to death is displayed in Fig. 3 a and b.

30-day mortality was 24.3% for the total cohort, 22.3% for “prone = no” and 26.4% for “prone = yes”. For patients with no information on proning 30-day mortality was 23.2%. Corresponding figures for 90-day mortality were 29.0% for the total cohort, 24.3 and 33.6 for “prone = no” and “prone = yes”, respectively. 90-day mortality for patients with no information on proning was 28.8 (Additional file 2: Table S1b).

For patients with PaO_2_/FiO_2_ ratio ≤ 20 kPa on ICU admission and receiving invasive mechanical ventilation within 24 h from ICU admission, 30-day mortality was 29.2% for the total cohort, 30.2% and 27.2% for “early prone = no and “early prone = yes”, respectively. The corresponding figures for 90-day mortality were 33.5% for the total cohort, 34.8% and 31.1% for “early prone = no and “early prone = yes”, respectively (Table 2.)

### Logistic regression estimating the association between initiation of early prone position ventilation and mortality

The regression analyses included only patients with PaO_2_/FiO_2_ ratio ≤ 20 kPa on ICU admission and receiving invasive mechanical ventilation within 24 h from ICU admission. On univariate logistic regression analysis of 30-day mortality, the odds ratio for “early prone = yes” compared to “early prone = no” was 0.86 (95% CI 0.69–1.08), and after adjustment the corresponding odds ratio was 0.92 (0.71–1.19) (Table 4).

**Table 4:** Univariate and multivariable logistic regression analysis for 30-day mortality

	Univariate		Multivariable	
	OR (95% CI)	P value	OR (95% CI)	P value
Prone				
Early = no	Reference		Reference	
Early = yes	0.86 (0.69—1.08)	0.2035	0.92 (0.71—1.19)	0.5206
Sex				
Women	Reference		Reference	
Men	1.33 (1.05—1.68)	0.0183	1.28 (0.99—1.67)	0.0645
Age, per year	1.07 (1.06—1.09)	< 0.001	1.07 (1.06—1.09)	< 0.001
Comorbidity				
Cardiac disease	1.93 (1.46—2.56)	< 0.001	1.14 (0.83—1.57)	0.4203
COPD/Asthma	1.08 (0.81—1.43)	0.5844	1.15 (0.84—1.56)	0.3846
Diabetes	1.22 (0.97—1.54)	0.0807	1.12 (0.86—1.45)	0.4021
Obesity^a^	0.63 (0.43—0.89)	0.0122	1.15 (0.76—1.72)	0.4979
Hypertension	1.33 (1.08—1.64)	0.0071	0.86 (0.67—1.10)	0.2276
Immune deficiency	1.52 (1.04—2.19)	0.0271	1.60 (1.05—2.42)	0.0279
Chronic liver disease	0.81 (0.18—2.72)	0.7502	0.57 (0.11—2.33)	0.4637
Chronic kidney disease	1.61 (1.05—2.44)	0.0268	1.05 (0.65—1.66)	0.8535
Neuromuscular disease	0.69 (0.20—1.94)	0.5167	0.62 (0.17—1.92)	0.4384
Malignancy^b^	3.02 (1.48—6.26)	0.0025	2.12 (0.97—4.69)	0.0585
PaO2/FiO2 ratio, per 1 kPa increase	0.97 (0.94—1)	0.0370	0.98 (0.95 – 1.02)	0.3362
SAPS3^c^, per 1 unit increase	1.05 (1.04—1.07)	< 0.001	1.05 (1.04—1.07)	< 0.001
Admission months				
March – April 2020	Reference		Reference	
May – August 2020	0.57 (0.39—0.83)	0.0037	0.45 (0.30—0.68	< 0.001
September – December 2020	1.19 (0.88—1.60)	0.2489	0.80 (0.57—1.11)	0.1848
January – April 2021	0.91 (0.71—1.18)	0.4793	0.62 (0.46—0.83)	0.0014

OR, Odds Ratio; CI, Confidence Interval; COPD, chronic obstructive pulmonary disease; PaO_2,_ arterial partial pressure of oxygen; FiO_2_, fraction of inspired oxygen; SAPS, simplified acute physiology score

^a^Obesity defined as BMI > 40 kg/m^2^

^b^Malignancy is defined as neoplasia spread beyond regional lymph nodes

^c^Recalculated after excluding age, comorbidities and PaO2/FiO2 ratio

aROC 0.749 for full model

The inverse probability of treatment weighted analysis revealed similar results (OR 0.97 (95% CI 0.92–1.02). Additional file 1: Fig. S2.

The odds ratios remained somewhat unchanged in the analyses restricted to patients with a) PaO_2_/FiO_2_ ≤ 13.3 kPa on ICU admission and b) patients still in treated in ICU within 48 h from ICU admission, Additional file 2: Tables S3 and S4. We also performed a logistic regression model of 90-day mortality with almost identical results, Additional file 2: Table S5.

## Discussion

In this nationwide study we describe the utilization of prone position in more than 6000 critically ill patient with COVID-19, where approximately half of the patients received prone position ventilation. Using a study-population of 1714 mechanically ventilated patients with respiratory insufficiency and hypoxemia we investigated the impact of early initiation of prone position on mortality. We found no association between early proning and survival. The crude 30-day mortality was 27.2% compared with 30.2% in patients not receiving early prone positioning.

After the striking results of the PROSEVA-study, recommended standard management of ARDS comprises of low tidal volumes, plateau pressures < 30 cm H_2_O and prone ventilation for moderate and severe ARDS with PaO_2_/FiO_2_ ≤ 13.3 kPa [10]. Adherence to an ARDS-protocol based on these parameters has been associated with improved survival [18]. Nevertheless, prone ventilation has been reported to be markedly underused with only 16.3% proning of severe ARDS and 5.5% of moderate ARDS patients in the LUNGSAFE study [3] and 32.9% and 10.3% for severe and moderate ARDS, respectively in the ARDS Prone Position Network (APRONET) study published two years later [2]. The most prominent reason for refraining prone ventilation was the apprehension that the oxygenation was not impaired enough or improving. Additional explanations included hemodynamic instability, deficient availability of competent staff and expected increase in workload [2].

During the COVID-19 pandemic the use of prone position for ARDS patients has increased considerably with a reported frequency ranging from 30% up to 75% [4, 13, 14], in line with the findings of the current study. This may reflect the limited treatment options for COVID-19 as well as increased adherence to current guidelines when hospitals were challenged with an overload of ARDS-patients. The overall use of prone position in this Swedish cohort is comparable to previous COVID-19 related ARDS studies; in total 47.3% and increasing over time during the pandemic. However, variation of utilization of prone position over time could hamper analyses of the impact of prone position on various patient outcomes.

In the current study, early initiation of prone position in a study-population of mechanically ventilated patients with PaO_2_/FiO_2_ ≤ 20 kPa had lower crude 30-day mortality compared to patients not receiving early initiation of prone position ventilation. This difference did not, however, reach statistical significance. Previous studies are relatively few and report varying results. In a study by Mathews et al., an increased 60-day survival was demonstrated in patients treated with prone ventilation within two days of ICU admission during the first wave of COVID-19 in the US [14]. Furthermore, a systematic review and metaanalysis on prone versus supine ventilation in COVID-19 patients reported an improved PaO_2_/FiO_2_ ratio with prone position and a lower mortality in patients that had been ventilated in prone position, although many small studies in the metaanalysis renders a low level of evidence level [19]. In addition, the Italian ICU-RER COVID-19 Collaboration demonstrated a correlation between oxygenation improvement due to prone positioning and ventilator-free days at 28 days and that non-responders had an increased mortality compared to responders[7]. On the contrary, an Italian multi-center study from the first COVID-19 wave with a high utilization of prone positioning could not demonstrate a survival benefit, rather a worse outcome mainly attributed to the higher disease severity in the proning group [13].

There are several possible explanatory factors for differences between previous results and the current study. In our study we only had data on PaO_2_/FiO_2_ ratio on ICU admission, thus we could not follow the dynamics in the severity of the patient condition after start of intensive care. Various definitions and modeling of admission period as a variable in analyses could also bias the results as optimal management of this variable is not clear. We included the admission period as a possible confounder in the analyses as both mortality and utilization of prone position have varied during the pandemic. In addition, and very important to point out, our study only investigates the effect of initiation of early prone position ventilation. Thus, patients in the group “early prone = no” could have been exposed to prone position after 24 h from start of invasive mechanical ventilation. Moreover, prone position may have been used during non-invasive ventilation before intubation and this could confound the results. In addition, the use of non-invasive ventilation before invasive ventilation increased markedly between COVID-19 periods and this may also have affected the results. To fully elucidate the impact of prone position ventilation in critically ill patients with COVID-19 a randomized controlled trial would be desirable. However, in consideration of the positive results from the PROSEVA trial, a randomized trial could be disputable from an ethical perspective.

As in previous studies on COVID-19, the predominant comorbidities were chronic hypertension, diabetes mellitus, COPD, cardiac disease and obesity [5, 20]. Most patients were admitted to the ICU from another hospital floor rather than the emergency department. A greater proportion of patients experienced a severe gas exchange disturbance with a PaO_2_/FiO_2_ ≤ 13 kPa (equivalent to severe ARDS) as compared to previous studies, largely explained by the inclusion criteria of PaO_2_/FiO_2_ $\le$ 20 kPa. The overall mortality of 29.5% in the cohort hence reflects a critically ill group of predominantly patients with a severe gas exchange disturbance. 

Our study has several strengths. We analyzed a large, nationwide multicenter cohort of ICU patients with COVID-19 with almost complete follow-up. All COVID-19 ICU patients in Sweden were eligible for inclusion, providing high generalizability to similar health-care systems. In addition, the inclusion period covered three peaks of the pandemic. SIR includes data on pre-ICU comorbid conditions, process of ICU care and long-term follow-up. Follow-up is ascertained by linkage between SIR and national registries. Furthermore, data in SIR are prospectively reported for quality-surveillance purposes and are therefore unbiased in relation to this study. 

We also identify several limitations, including the observational and retrospective study design. It is possible that important data related to the outcome are lacking. We had no data on socioeconomic status nor ethnicity. Data on process of care before ICU admission are not included in SIR. Approximately one-third (31.1%) of the total study cohort had no data on PaO_2_/FiO_2_ ratio on ICU admission. A majority of these patients did not receive any non-invasive or invasive mechanical ventilation on admission and 30-day mortality was 19.8% compared with 29.2% for patients with PaO_2_/FiO_2_ ratio ≤ 20 kPa on ICU admission and receiving invasive mechanical ventilation within 24 h from ICU admission. Data on criteria for initiation and terminalization of the proning sessions were unfortunately lacking. Information on ventilator settings, duration and the number of proning sessions and contraindications to prone position would have added value to this study, but these data were not available, nor was data on the dynamics of PaO_2_/FiO_2_ ratio. Clinical data, e.g. pulmonary compliance, physiological response to prone position and imaging would have added important information but was unfortunately not available. Furthermore, due to the rapid and persistent increase in the need of ICU beds, ICU recourses were of course strained during the study period. The global cumulative knowledge concerning treatment of COVID-19 and treatment algorithms are constantly evolving. Treatment with high-dose low molecular weight heparins is not included in SIR, nor is the use of steroids. Finally, it is possible that the registration of prone position in SIR in some cases also could include patients only partially proned i.e., placed in the side position.

## Conclusions

In this nationwide cohort study of more than 6000 ICU COVID-19 patients covering several pandemic peaks, almost half of the patients were treated with prone position ventilation. The use of early prone position treatment increased markedly over time in patients with severe hypoxemia. We found no association between early use of prone positioning and survival in patients on mechanical ventilation with severe hypoxemia on ICU admission. The crude 30-day mortality was 27.2% compared to 30.2% in patients not receiving early prone positioning. To fully elucidate the effect and timing of prone position ventilation in critically ill patients with COVID-19 further studies are desirable.

## Supplementary Information

**Additional file 1.** **Figure S1**. Loess smoothed plot of use of early prone position over time. **Figure S2**. Inverse probability of treatment weighting analysis.

**Additional file 2. Table S1a**. Patient Characteristics. **Table S1b**. ICU parameters. **Table S2**. Organ support therapies. **Table S3**. Univariate and multivariable logistic regression analysis for 30-day mortality in severe ARDS (PaO2/FiO_2_≤13.3 kPa), 1187 patients. **Table S4**. Univariable and multivariable logistic regression analysis for 30-day mortality length of stay > 48h, 1379 patients. **Table S5**. Univariate and multivariable logistic regression analysis for 90-day mortality. **Table S6**. Comparison between COVID-19 periods for the study-population with low oxygenation and early mechanical ventilation.

## Data Availability

The data that support the findings of this study are available from SIR but restrictions apply to the availability of these data, which were used under license for the current study, and so are not publicly available. Data are however available from the authors upon reasonable request and with permission of SIR.
