# Supplementary material for: Prevalence and impact of early prone position on 30-day mortality in mechanically ventilated patients with COVID-19: a nationwide cohort study
Source: Crit Care. 2022 Sep 4;26:264. doi: 10.1186/s13054-022-04122-w (PMC9441133; doi:10.1186/s13054-022-04122-w)
Supplement: Supplementary file 1 — Additional file 1. Figure S1. Loess smoothed plot of use of early prone position over time. Figure S2. Inverse probability of treatment weighting analysis. [file 13054_2022_4122_MOESM1_ESM.docx]

**Additional file 1: Figure S1. Loess smoothed plot of use of early prone position over time.**

Blue line = mean, shaded area = 95% confidence interval

**Additional file 1: Figure S2. Inverse probability of treatment weighting analysis**

Standardised mean difference for the predictors included in the logistic regression models between early “prone = yes” and “early prone = no”, before and after weighting.
