# Supplementary material for: Prevalence and impact of early prone position on 30-day mortality in mechanically ventilated patients with COVID-19: a nationwide cohort study
Source: Crit Care. 2022 Sep 4;26:264. doi: 10.1186/s13054-022-04122-w (PMC9441133; doi:10.1186/s13054-022-04122-w)
Supplement: Supplementary file 2 — Additional file 2. Table S1a. Patient Characteristics. Table S1b. ICU parameters. Table S2. Organ support therapies. Table S3. Univariate and multivariable logistic regression analysis for 30-day mortality in severe ARDS (PaO2/FiO2≤13.3 kPa), 1187 patients. Table S4. Univariable and multivariable logistic regression analysis for 30-day mortality length of stay > 48h, 1379 patients. Table S5. Univariate and multivariable logistic regression analysis for 90-day mortality. Table S6. Comparison between COVID-19 periods for the study-population with low oxygenation and early mechanical ventilation. [file 13054_2022_4122_MOESM2_ESM.docx]

**Additional file 2: Table S1a. Patient Characteristics**

|  | **No. (%)** | | | |
| --- | --- | --- | --- | --- |
|  | **All patients** | **Prone = NO** | **Prone =YES** | **Prone = UNKNOWN** |
| ***Patient demographics, characteristics and comorbidities at ICU admission*** | | | |  |
| No. (%) | 6350 | 2949 (46.4) | 3008 (47.4) | 393 (6.2) |
| Women | 1843 (29.0) | 928 (31.5) | 812 (27.0) | 103 (26.2) |
| Age, median (IQR), y | 64 (54-72) | 64 (53-73) | 64 (55-71) | 64 (56-72) |
| Age, Interval, y |  |  |  |  |
| <40 | 379 (6.0) | 227 (7.7) | 131 (4.4) | 21 (5.3) |
| 40-49 | 636 (10.0) | 317 (10.7) | 285 (9.5) | 34 (8.7) |
| 50-59 | 1379 (21.7) | 601 (20.4) | 692 (23.0) | 86 (21.9) |
| 60-69 | 1907 (30.0) | 810 (27.5) | 976 (32.4) | 121 (30.8) |
| 70-79 | 1677 (26.4) | 765 (25.9) | 802 (26.7) | 110 (28.0) |
| ≥80 | 372 (5.9) | 229 (7.8) | 122 (4.1) | 21 (5.3) |
| Admission month |  |  |  |  |
| March – April 2020 | 1409 (22.2) | 675 (22.9) | 630 (20.9) | 104 (26.5) |
| May – August 2020 | 915 (14.4) | 415 (14.1) | 438 (14.6) | 62 (15.8) |
| September – December 2020 | 1439 (22.7) | 704 (23.9) | 662 (22) | 73 (18.6) |
| January – April 2021 | 2587 (40.7) | 1155 (39.2) | 1278 (42.5) | 154 (39.2) |
| Location before ICU admission |  |  |  |  |
| Emergency department | 1421 (22.4) | 660 (22.4) | 662 (22.0) | 99 (25.2) |
| Hospital floor | 4929 (77.6) | 2289 (77.6) | 2346 (78.0) | 294 (74.8) |
| Time from symptom to ICU admission, median (IQR), d |  |  |  |  |
| No. with data | 6260 | 2896 | 2980 | 354 |
| Median (IQR), d | 10 (7-13) | 10 (7-13) | 10 (7-13) | 9 (7-12) |
| Hospital level |  |  |  |  |
| Tertiary | 2251 (35.4) | 1008 (34.2) | 1054 (35) | 189 (48.1) |
| County | 3187 (50.2) | 1523 (51.6) | 1498 (49.8) | 166 (42.2) |
| Local | 912 (14.4) | 418 (14.2) | 456 (15.2) | 38 (9.7) |
| Days at hospital before ICU admission, median (IQR), d | 2 (0-4) | 2 (0-4) | 2 (0-4) | 1 (0-4) |
| Pregnant. No. | 48 | 33 | 13 | 2 |
| Comorbidities |  |  |  |  |
| None | 1765 (27.8) | 803 (27.2) | 850 (28.3) | 112 (28.5) |
| One or more | 4585 (72.2) | 2146 (72.8) | 2158 (71.7) | 281 (71.5) |
| Chronic hypertension | 3063 (48.2) | 1376 (46.7) | 1496 (49.7) | 191 (48.6) |
| Chronic cardiac disease | 1096 (17.3) | 543 (18.4) | 480 (16.0) | 73 (18.6) |
| COPD/Asthma | 1171 (18.4) | 549 (18.6) | 560 (18.6) | 62 (15.8) |
| Immune deficiency | 550 (8.7) | 259 (8.8) | 260 (8.6) | 31 (7.9) |
| Chronic liver disease | 59 (0.9) | 29 (1.0) | 25 (0.8) | 5 (1.3) |
| Chronic kidney disease | 416 (6.6) | 225 (7.6) | 166 (5.5) | 25 (6.4) |
| Diabetes | 1711 (26.9) | 810 (27.5) | 807 (26.8) | 94 (23.9) |
| Neuromuscular disease | 102 (1.6) | 58 (2.0) | 35 (1.2) | 9 (2.3) |
| Obesity^a^ | 578 (9.1) | 277 (9.4) | 261 (8.7) | 40 (10.2) |
| Malignancy^b^ | 142 (2.2) | 75 (2.5) | 60 (2.0) | 8 (2.0) |

Abbreviations: IQR, interquartile range; y: years; d: days; ICU, intensive care unit; COPD, chronic obstructive pulmonary disease

^a^Obesity is defined as BMI >40kg/m^2^, ^b^Malignancy is defined as neoplasia spread beyond regional lymph nod

**Additional file 2: Table S1b. ICU parameters**

| ***Ventilatory parameters at ICU admission (within one hour before until one hour after admission)*** | | | | |
| --- | --- | --- | --- | --- |
| PaO_2_, kPa. Median (IQR) |  |  |  |  |
| No. with data | 5641 | 2645 | 2673 | 323 |
| Median (IQR) | 8.7 (7.6-10.2) | 9.0 (7.8-10.6) | 8.5 (7.5-9.8) | 8.7 (7.4-10.1) |
| FiO_2_, %. Median (IQR) |  |  |  |  |
| No. with data | 4408 | 2059 | 2126 | 223 |
| Median (IQR) | 75 (60-90) | 70 (60-85.5) | 80 (65-95) | 80 (60-85) |
| PaO_2_/FiO_2_ ratio. Median (IQR) |  |  |  |  |
| No. with data | 4372 | 2039 | 2110 | 223 |
| Median (IQR) | 12.1 (9.3-16.1) | 13.2 (10.0-17.7) | 11.3 (8.8-14.7) | 12.3 (9.4-16.3) |
| PaO_2_/FiO_2_ ratio categories. kPa |  |  |  |  |
| >26.6 - ≤40.0 | 148 (3.4) | 101 (5.0) | 39 (1.8) | 8 (3.6) |
| >13.3 - ≤26.6 | 1593 (36.4) | 842 (41.3) | 669 (31.7) | 82 (36.8) |
| ≤13.3 | 2550 (58.3) | 1038 (50.9) | 1385 (65.6) | 127 (57.0) |
| ***Treatment and vital signs at ICU admission (within one hour before until one hour after admission)*** | | | | |
| Vasopressor on admission, No (%) | 214 (3.4) | 108 (3.7) | 97 (3.2) | 9 (2.3) |
| Body temperature |  |  |  |  |
| No with data | 6027 | 2807 | 2883 | 337 |
| Degrees Celsius | 37.6 (37.0-38.3) | 37.5 (37-38.2) | 37.7 (37.0-38.4) | 37.5 (37.0-38.3) |
| Fever^a^, No (%) | 1938 (32.2) | 818 (29.1) | 1016 (35.2) | 104 (30.9) |
| Systolic blood pressure, min, mmHg |  |  |  |  |
| No. with data | 6137 | 2858 | 2920 | 359 |
| Median (IQR) | 120 (105-140) | 120 (105-140) | 120 (106-140) | 120 (103-140) |
| Heart rate, maximum, beats/min |  |  |  |  |
| No. with data | 6208 | 2884 | 2950 | 374 |
| Median (IQR) | 91 (80-107) | 90 (80-107) | 92 (80-107) | 95 (84-110) |
| ***Laboratory findings at ICU admission (within one hour before until one hour after admission)*** | | | | |
| White blood cell count, x10^9^/L |  |  |  |  |
| No. with data | 5822 | 2686 | 2792 | 344 |
| Median (IQR) | 8.6 (6.5-12.0) | 8.8 (6.4-12.0) | 8.9 (6.5-12.1) | 8.8 (6.6-12.0) |
| pH |  |  |  |  |
| No. with data | 6063 | 2796 | 2895 | 372 |
| Median (IQR) | 7.45 (7.40-7.48) | 7.45 (7.40-7.48) | 7.45 (7.40-7.48) | 7.45 (7.40-7.48) |
| Creatinine, mg/dL |  |  |  |  |
| No. with data | 5844 | 2705 | 2789 | 350 |
| Median (IQR) | 71 (58-94) | 71 (57-97) | 71 (58-92) | 73 (57-98) |
| Bilirubin, mg/dL |  |  |  |  |
| No. with data | 5629 | 2602 | 2693 | 334 |
| Median (IQR) | 9 (6-12) | 9 (6-12) | 9 (6-12) | 9 (6-12) |
| ***Scores*** | | | | |
| SAPS3 at admission, median (IQR) | 55 (48-61) | 54 (48-61) | 55 (49-62) | 55 (49-61) |
| Predicted risk of death (SAPS3), median (IQR), % | 12 (6-21) | 11 (6-21) | 12 (6-22) | 12 (6-21) |

Abbreviations: IQR, interquartile range; y: years; d: days; ICU, intensive care unit; PaO_2,_ arterial partial pressure of oxygen; FiO_2_, fraction of inspired oxygen; SAPS, simplified acute physiology score. ^a^Fever is defined as body temperature above 38 ^o^C

**Additional file 2: Table S2. Organ support therapies**

|  | **No (%)** | | | |
| --- | --- | --- | --- | --- |
| **Variable** | **All**  **(n = 6350)** | **Prone = NO**  **(n = 2949)** | **Prone =YES**  **(n = 3008)** | **Prone = UNKNOWN**  **(n = 393)** |
| Invasive mechanical ventilation, No (%) | 4174 (65.7) | 1308 (44.4) | 2560 (85.1) | 307 (78.1) |
| Duration of invasive mechanical ventilation, median (IQR), h | 279 (148-490) | 189 (99-245) | 333 (191-566) | 341 (169-545) |
| Renal replacement therapy, No./total (%) | 772/5715 (13.5) | 193/2714 (7.1) | 519/2670 (19.4) | 60/331 (18.1) |
| ECMO, No./total (%) | 55/3399 (1.6) | 9/1777 (0.5) | 43/1586 (2.7) | 3/36 (8.3) |
| Tracheostomy, No. (%) | 1562 (24.6) | 315 (10.7) | 1119 (37.2) | 128 (32.6) |
| More than admission^a^ | 1748 (27.5) | 467 (15.8) | 1096 (36.4) | 185 (47.1) |
| ICU length of stay, median (IQR), d | 10 (4-19) | 5 (2-11) | 15 (9-26) | 13 (7-23) |
| ***Outcome*** | | | | |
| 30-day mortality. No. (%) | 1542 (24.3) | 657 (22.3) | 794 (26.4) | 91 (23.2) |
| 90-day mortality. No. (%) | 1841 (29.0) | 717 (24.3) | 1011 (33.6) | 113 (28.8) |

Abbreviations: IQR, interquartile range; ECMO, extracorporeal membrane oxygenation; h, hours; ICU, intensive care unit; d, days

^a^Most often due to optimization of ICU resources

**Additional file 2: Table S3. Univariate and multivariable logistic regression analysis for 30-day mortality in severe ARDS (PaO_2_/FiO_2_≤13.3 kPa), 1187 patients**

|  | **Univariate** | | **Multivariable^a^** | |
| --- | --- | --- | --- | --- |
|  | **OR (95% CI)** | **P value** | **OR (95% CI)** | **P value** |
| Early prone | 0.87 (0.67 - 1.12) | 0.2699 | 0.96 (0.72 - 1.28) | 0.7952 |
| Sex |  |  |  |  |
| Women | Reference |  | Reference |  |
| Men | 1.5 (1.14 – 2) | 0.0043 | 1.47 (1.08 - 2.02) | 0.0161 |
| Age, per year | 1.07 (1.06 - 1.08 ) | <0.001 | 1.07 (1.06 - 1.09) | <0.001 |
| Comorbidity |  |  |  |  |
| Cardiac disease | 2.26 (1.61 - 3.17) | <0.001 | 1.38 (0.94 - 2.01) | 0.0996 |
| COPD/Asthma | 1.05 (0.76 - 1.46) | 0.7524 | 1.17 (0.81 - 1.67) | 0.3932 |
| Diabetes | 1.37 (1.05 - 1.78) | 0.0185 | 1.31 (0.97 - 1.77) | 0.0804 |
| Obesity^a^ | 0.69 (0.44 - 1.03) | 0.0799 | 1.19 (0.74 - 1.9) | 0.4621 |
| Hypertension | 1.27 (0.99 - 1.63) | 0.0575 | 0.76 (0.56 - 1.02) | 0.0678 |
| Immune deficiency | 2.08 (1.35 - 3.19) | <0.001 | 2.22 (1.35 - 3.63) | 0.0015 |
| Chronic liver disease | 0.58 (0.09 - 2.32) | 0.4894 | 0.41 (0.05 - 2.08) | 0.3303 |
| Chronic kidney disease | 1.96 (1.21 - 3.14) | 0.0057 | 1.43 (0.83 - 2.45) | 0.1939 |
| Neuromuscular disease | 1.03 (0.28 - 3.19) | 0.9584 | 1.19 (0.29 - 4.18) | 0.7974 |
| Malignancy^b^ | 2.6 (1.09 - 6.3) | 0.0303 | 1.97 (0.76 - 5.13) | 0.1586 |
| SAPS3, per 1 unit increase^c^ | 1.05 (1.03 - 1.07) | <0.001 | 1.04 (1.02 - 1.06) | <0.001 |
| PaO_2_/FiO_2_ | 0.94 (0.88 - 0.99) | 0.0259 | 0.93 (0.88 - 1) | 0.0417 |
| Admission month |  |  |  |  |
| March-April | Reference |  | Reference |  |
| May-Aug | 0.56 (0.35 - 0.88) | 0.0131 | 0.42 (0.26 - 0.69) | 0.0006 |
| Sept-Dec | 1.18 (0.82 - 1.7) | 0.3787 | 0.7 (0.46 - 1.07) | 0.0995 |
| Jan-May | 1 (0.73 - 1.35) | 0.9745 | 0.64 (0.45 - 0.91) | 0.0121 |

Abbreviations: OR, Odds Ratio; CI, Confidence Interval; COPD, chronic obstructive pulmonary disease; PaO_2,_ arterial partial pressure of oxygen; FiO_2_, fraction of inspired oxygen; SAPS, simplified acute physiology score.

^a^Obesity is defined as BMI >40kg/m^2^

^b^Malignancy is defined as neoplasia spread beyond regional lymph nodes

^c^Recalculated after excluding age and comorbidities

aROC 0.744 for full model.

**Additional file 2: Table S4. Univariable and multivariable logistic regression analysis for 30-day mortality length of stay > 48h, 1379 patients**

|  | **Univariate** | | **Multivariable^a^** | |
| --- | --- | --- | --- | --- |
|  | **OR (95% CI)** | **P value** | **OR (95% CI)** | **P value** |
| Early prone | 0.87 (0.68 - 1.1) | 0.2472 | 0.84 (0.64 - 1.11) | 0.2211 |
| Sex |  |  |  |  |
| Women | Reference |  | Reference |  |
| Men | 1.49 (1.15 - 1.96) | 0.0031 | 1.43 (1.07 - 1.93) | 0.0164 |
| Age, per year | 1.08 (1.06 - 1.09) | <0.001 | 1.08 (1.06 - 1.09) | <0.001 |
| Comorbidity |  |  |  |  |
| Cardiac disease | 1.94 (1.42 - 2.65) | 0.0000 | 1.18 (0.83 - 1.67) | 0.3533 |
| COPD/Asthma | 1.01 (0.73 - 1.38) | 0.9477 | 1.04 (0.73 - 1.46) | 0.8302 |
| Diabetes | 1.31 (1.02 - 1.69) | 0.0365 | 1.13 (0.84 - 1.5) | 0.4170 |
| Obesity^a^ | 0.56 (0.36 - 0.83) | 0.0056 | 1 (0.63 - 1.56) | 0.9931 |
| Hypertension | 1.38 (1.1 - 1.75) | 0.0064 | 0.86 (0.65 - 1.14) | 0.2940 |
| Immune deficiency | 1.51 (1.01 - 2.25) | 0.0437 | 1.75 (1.11 - 2.73) | 0.0144 |
| Chronic liver disease | 0.61 (0.09 - 2.43) | 0.5270 | 0.44 (0.06 - 2.13) | 0.3500 |
| Chronic kidney disease | 1.41 (0.87 - 2.25) | 0.1517 | 0.99 (0.58 - 1.65) | 0.9587 |
| Neuromuscular disease | 0.81 (0.22 - 2.34) | 0.7137 | 0.84 (0.22 - 2.71) | 0.7876 |
| Malignancy^b^ | 2.9 (1.33 - 6.44) | 0.0074 | 2.1 (0.91 - 4.91) | 0.0815 |
| SAPS3, per 1 unit increase^c^ | 1.04 (1.03 - 1.06) | <0.001 | 1.03 (1.02 - 1.05) | <0.001 |
| PaO_2_/FiO_2_ | 0.95 (0.92 - 0.98) | 0.0024 | 0.95 (0.92 - 0.99) | 0.0088 |
| Admission month |  |  |  |  |
| March-April | Reference |  | Reference |  |
| May-Aug | 0.68 (0.44 - 1.02) | 0.0673 | 0.56 (0.35 - 0.88) | 0.0134 |
| Sept-Dec | 1.18 (0.85 - 1.64) | 0.3276 | 0.79 (0.54 - 1.15) | 0.2219 |
| Jan-May | 0.95 (0.72 - 1.26) | 0.7080 | 0.66 (0.48 - 0.92) | 0.0143 |

Abbreviations: OR, Odds Ratio; CI, Confidence Interval; COPD, chronic obstructive pulmonary disease; PaO_2,_ arterial partial pressure of oxygen; FiO_2_, fraction of inspired oxygen; SAPS, simplified acute physiology score.

^a^Obesity is defined as BMI >40kg/m^2^

^b^Malignancy is defined as neoplasia spread beyond regional lymph nodes

^c^Recalculated after excluding age and comorbidities

aROC 0.748 for full model.

**Additional file 2: Table S5. Univariate and multivariable logistic regression analysis for 90-day mortality**

|  | **Univariate** | | **Multivariable^a^** | |
| --- | --- | --- | --- | --- |
|  | **OR (95% CI)** | **P value** | **OR (95% CI)** | **P value** |
| Early prone | 0.84 (0.68 - 1.05) | 0.1228 | 0.89 (0.69 - 1.14) | 0.3544 |
| Sex |  |  |  |  |
| Women | Reference |  | Reference |  |
| Men | 1.39 (1.11 - 1.75) | 0.0041 | 1.34 (1.04 - 1.74) | 0.0226 |
| Age, per year | 1.07 (1.06 - 1.09) | <0.001 | 1.08 (1.06 - 1.0) | <0.001 |
| Comorbidity |  |  |  |  |
| Cardiac disease | 2.17 (1.65 - 2.87) | 0.0000 | 1.3 (0.95 - 1.77) | 0.1032 |
| COPD/Asthma | 1.14 (0.87 - 1.49) | 0.3473 | 1.21 (0.9 - 1.64) | 0.2028 |
| Diabetes | 1.28 (1.03 - 1.6) | 0.0259 | 1.17 (0.91 - 1.51) | 0.2236 |
| Obesity^a^ | 0.67 (0.47 - 0.94) | 0.0219 | 1.25 (0.84 - 1.83) | 0.2654 |
| Hypertension | 1.37 (1.12 - 1.68) | 0.0020 | 0.86 (0.67 - 1.09) | 0.2202 |
| Immune deficiency | 1.5 (1.04 - 2.15) | 0.0286 | 1.62 (1.07 - 2.44) | 0.0220 |
| Chronic liver disease | 0.99 (0.26 - 3.16) | 0.9874 | 0.74 (0.17 - 2.83) | 0.6731 |
| Chronic kidney disease | 1.48 (0.97 - 2.24) | 0.0625 | 0.93 (0.58 - 1.48) | 0.7610 |
| Neuromuscular disease | 0.99 (0.34 - 2.56) | 0.9846 | 0.92 (0.29 - 2.67) | 0.8862 |
| Malignancy^b^ | 3.21 (1.57 - 6.84) | 0.0017 | 2.26 (1.03 - 5.12) | 0.0448 |
| SAPS3, per 1 unit increase^c^ | 1.05 (1.04 - 1.08) | <0.001 | 1.05 (1.04 - 1.08) | <0.001 |
| PaO_2_/FiO_2_ | 0.97 (0.95 - 1) | 0.0443 | 0.99 (0.95 - 1.02) | 0.3572 |
| Admission month |  |  |  |  |
| March-April | Reference |  | Reference |  |
| May-Aug | 0.57 (0.39 - 0.80) | 0.0017 | 0.44 (0.30 - 0.65) | <0.001 |
| Sept-Dec | 1.18 (0.88 - 1.57) | 0.2593 | 0.79 (0.57 - 1.1) | 0.1631 |
| Jan-May | 0.88 (0.69 - 1.13) | 0.3264 | 0.6 (0.45 - 0.8) | <0.001 |

Abbreviations: OR, Odds Ratio; CI, Confidence Interval; COPD, chronic obstructive pulmonary disease; PaO_2,_ arterial partial pressure of oxygen; FiO_2_, fraction of inspired oxygen; SAPS, simplified acute physiology score.

^a^Obesity is defined as BMI >40kg/m^2^

^b^Malignancy is defined as neoplasia spread beyond regional lymph nodes

^c^Recalculated after excluding age, oxygenation and comorbidities

aROC 0.753 for full model.

**Additional file 2: Table S6. Comparison between COVID-19 periods for the study-population with low oxygenation and early mechanical ventilation**

| **Period** | **2020** | **2021** |
| --- | --- | --- |
| Patients | 1068 | 646 |
| Early prone n (%) | 277 (25.9) | 293 (45.4) |
| Duration of invasive mechanical ventilation, median (IQR) h | 300(174 – 497) | 215 (113 – 387) |
| Renal replacement therapy, No./total (%) | 232/1017 (22.8) | 80/621 (12.9) |
| ECMO, No./total (%) | 16/675(2.4) | 7/307(2.3) |
| Tracheostomy, No. (%) | 382/1068(35.8) | 196/646(30.3) |
| More than admission^a^ | 349/1068(32.7) | 227/646(35.1) |
| ICU length of stay, median (IQR), d | 15(9-24) | 12(7-19) |
| Delay from arrival at ICU to invasive mechanical ventilation, h, median(IQR) | 1.00(0.33 – 4.42) | 1.32 (0.50 – 6.04) |
| Non-invasive ventilation before invasive ventilation, n(%) | 172 /955 (18.0) | 192/558 (35.0) |

Abbreviations: IQR, interquartile range; ECMO, extracorporeal membrane oxygenation; h, hours; ICU, intensive care unit; d, days,  ^a^Most often due to optimization of ICU resources
